# Predicting domain-domain interaction based on domain profiles with feature selection and support vector machines

**DOI:** 10.1186/1471-2105-11-537

**Published:** 2010-10-29

**Authors:** Alvaro J González, Li Liao

**Affiliations:** 1 Department of Computer and Information Sciences, University of Delaware 421 Smith Hall, Newark, DE 19716, USA

## Abstract

**Background:**

Protein-protein interaction (PPI) plays essential roles in cellular functions. The cost, time and other limitations associated with the current experimental methods have motivated the development of computational methods for predicting PPIs. As protein interactions generally occur via domains instead of the whole molecules, predicting domain-domain interaction (DDI) is an important step toward PPI prediction. Computational methods developed so far have utilized information from various sources at different levels, from primary sequences, to molecular structures, to evolutionary profiles.

**Results:**

In this paper, we propose a computational method to predict DDI using support vector machines (SVMs), based on domains represented as interaction profile hidden Markov models (ipHMM) where interacting residues in domains are explicitly modeled according to the three dimensional structural information available at the Protein Data Bank (PDB). Features about the domains are extracted first as the Fisher scores derived from the ipHMM and then selected using singular value decomposition (SVD). Domain pairs are represented by concatenating their selected feature vectors, and classified by a support vector machine trained on these feature vectors. The method is tested by leave-one-out cross validation experiments with a set of interacting protein pairs adopted from the 3DID database. The prediction accuracy has shown significant improvement as compared to *InterPreTS *(Interaction Prediction through Tertiary Structure), an existing method for PPI prediction that also uses the sequences and complexes of known 3D structure.

**Conclusions:**

We show that domain-domain interaction prediction can be significantly enhanced by exploiting information inherent in the domain profiles via feature selection based on Fisher scores, singular value decomposition and supervised learning based on support vector machines. Datasets and source code are freely available on the web at http://liao.cis.udel.edu/pub/svdsvm. Implemented in Matlab and supported on Linux and MS Windows.

## Background

Protein-protein interaction (PPI) plays essential roles in cellular functions. With the emerging new paradigm of systems biology, much of the research focus has been shifted from studying individual proteins and their functions to studying how they interact with each other, forming biological networks in fulfilling cellular processes. Great advancements have been witnessed in experimental technologies, such as yeast two-hybrid (Y2H) and coimmunoprecipitation (CoIP), for detecting PPIs [1]. Still, the cost, time and other limitations associated with the current experimental methods have motivated development of computational methods for predicting PPIs.

Over the past few years, many computational methods have been developed for PPI prediction, utilizing information from various sources at different levels, from primary sequences [2-4], to molecular structures [5-7], to evolutionary profiles [8-13], to microarray expression data [14], to networks information [15,16]. In general, more sensitive prediction tends to require extensive information, *e.g*., phylogenetic information, and more specific prediction tends to require more detailed information, *e.g*., the structural information. The 3D structure of proteins plays an important role in PPI; as proteins interact with one another, their structures need to match, *i.e*. the interacting domains (interfaces) must be folded into certain conformations so that they attract each other (energetically and physically). For example, the interface at one protein that has a concave shape tends to require a convex shape for its interacting partner. Various constraints at the interface may in turn impose constraints on the amino acid composition at the interfaces. Therefore, identifying protein interaction at the domain level can serve as an important intermediate step toward an effective approach for prediction of PPI [17-22], even though the inference from pairwise DDI to PPI can be complicated due to factors like the presence of multi-domains, *e.g*., combination of domains may block interactions that are otherwise suggested if solely based on individual domain interactions [23].

General domain identification itself is a highly non-trivial task. Sequence patterns based on such compositions typically lack enough uniqueness to be solely relied upon for domain identification. In fact, multiple sequence alignments of proteins that are known to contain the same domain show variations in sequence composition. Hidden Markov models (HMM) are among the most successful efforts to capture the commonalities of a given domain while allowing variations. A collection of HMMs covering many common protein domains and families is available in the Pfam database [24].

For interface domains, the interaction sites impose strong constraints and therefore play a key role in identifying the domains. However, interaction site information is not readily available for many proteins and the dataset of PPIs that have been resolved using crystallography remains relatively small. To tackle this issue, Friedrich and coworkers developed a new method, called interaction profile hidden Markov model (ipHMM), which modifies the ordinary profile hidden Markov model (pHMM) by adding to the model architecture new states explicitly representing residues on the interface [25]. This leads to improved accuracy in interaction domain identification.

Despite the improvement, the membership of domain families is still established at the best via probabilistic modeling and therefore false positives and negatives are not uncommon. As shown in detail in section 1, the error rates for identification of individual domains will multiply when used for predicting domain-domain interactions (DDIs). More seriously, although supported by other evidences such as domain modularity of proteins and shared DDIs among PPIs, in most cases experimental verification in support of the DDI-PPI correspondence is still missing [13].

In this work, we propose a new computational method to address these issues, in particular by extracting and selecting features encoded in the interaction domain profiles for DDI prediction. The method is based on a framework first proposed by Jaakkola *et.al*., which combines generative models and discriminative classifiers in tandem [26,27]. In our case, the generative model is the ipHMM representing domains that, based on the structural information, are known to be involved in PPI (this means the training data is partly based on the structural information). Once an ipHMM is trained, it can be used to predict domains and interaction sites for proteins with only the sequential information as input. To mitigate the above-mentioned multiplying effect of false domain prediction on DDI prediction, the learning of interactions is deferred to a discriminative classifier, which is a support vector machine (SVM) in our case.

This two-stage framework allows us to do sophisticated feature extraction and selection from the domain profiles. For feature extraction, we represent proteins by vectors that are composed of the Fisher scores, which are defined as the gradient, with respect to the model parameters, of the posterior probability when the proteins are aligned with the ipHMM. Because of the large number of parameters for a typical ipHMM and the fact that not all parameters play equal roles in determining a protein's membership to the domain family described by the model, feature selection is necessary and essential. Feature selection in this work is based on the singular value decomposition (SVD) technique. Protein pairs are represented by concatenated feature vectors, which are used to train the SVM classifier. The method is tested by leave-one-out (LOO) cross validation experiments with a set of interacting protein pairs adopted from the 3DID database [28], and the prediction accuracy is at about 0.90 measured by ROC score with significantly higher confidence as compared with a similar method.

## 1 Methods

In this section, we first introduce the interaction profile hidden Markov models that are used to capture domain properties including the structural information. We then describe how to calculate the Fisher score vector for a protein whose sequence is aligned to an ipHMM. We end the section with the feature selection scheme based on SVD.

**Figure 1:**
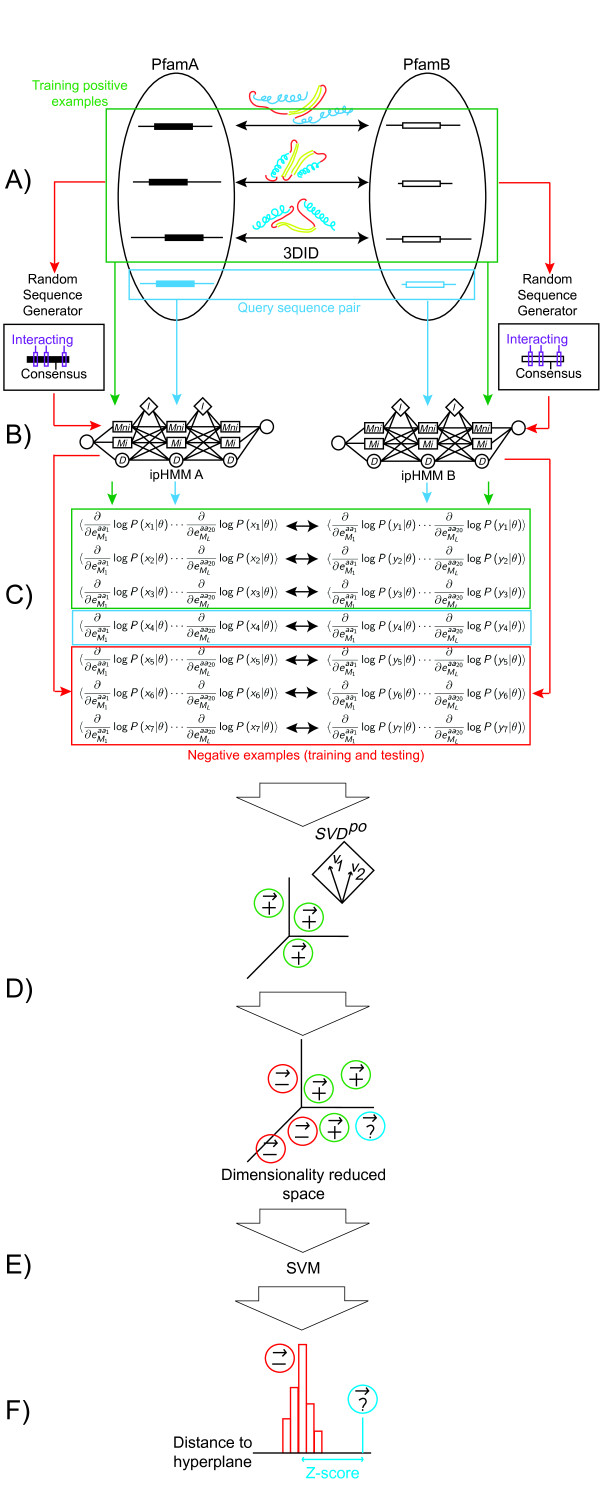
**Flow chart of the method**. Panel A shows a pair of interacting domain families, PfamA and PfamB, where for protein pairs across families, 3D structures are known that confirm interaction. The consensus sequence of each alignment as well as the set of interacting positions are used to produce random sequences from each family. Panel B shows the architecture of two ipHMMs, containing interacting states (interface residues) marked as *M_i_*, and non-interacting as *M_ni _*. Each protein sequence (positive or negative) is aligned to its corresponding ipHMM, and the sufficient statistics of this  alignment are used to characterize the sequence by means of Fisher vectors (panel C). In panel D, feature selection is calculated for the entire dataset using *SVD^PO^*, and it shows how all the dimensionality reduced vectors can be placed in the same vector space, which leaves them ready for training a support vector machine (SVM). The blue box shows a query sequence pair, where each of the proteins aligns to one of the domain families. Random negative examples are generated again, but now to be used in testing. Panels B, C and D work the same way as in training. In panel E, the SVM is used for classifying test examples. All distances to the hyperplane form a histogram (panel F), where the query sequence, if it is an actual interacting pair, is expected to have a large Z-score.

The overall design is illustrated in the flow diagram of Figure 1. Panel A shows two domain families, PfamA and PfamB, with member proteins retrieved from the Pfam database [24]. Some member proteins in PfamA and PfamB are known to be interacting based on structural information collected from the 3DID database [28]. These proteins are paired as indicated by the double arrows, and are used as training (positive) examples to construct ipHMMs for PfamA and PfamB respectively. As shown in panel B, the architecture of the ipHMM contains new states marked as *M_i _*to describe residues that are on the interacting interface. All member proteins in each family are then aligned to their ipHMM and the Fisher scores are computed as described in subsection Fisher Scores. As a result, proteins are now represented as Fisher score vectors, and protein pairs are represented by concatenating their individual vectors, as shown in panel C. Interacting pairs (positive) and non-interacting pairs (negative) are prepared for training and testing the SVM, as described in subsection Preparation of training and testing sets. The negative examples are mostly random sequences generated from the model consensus as described in subsection Negative examples, except for one case where homologous family members are known to be negative, as studied in subsection Case study of real negative examples. In the next step, as shown in panel D, SVD is carried out for positive training examples, and the subspace *SVD^PO^*, spanned by the positive singular vectors that make up for 80% of the variance in the training set, are used for feature selection. In panel E, after projecting all examples onto *SVD^PO ^*we obtain a training dataset made of vectors of reduced dimension with selected features. This dataset is used to train a support vector machine. Through the pipeline, a sequence pair, shown in Panel A as query, along with some negative examples, are reserved for testing, using the trained SVM (Panel E). In panel F, the distance of each test example to the hyperplane of the SVM is plotted in a histogram, and the Z-score of the query pair is used to predict a potential interaction. All these steps are detailed in the following subsections.

### Interaction Profile Hidden Markov Models

**Figure 2:**
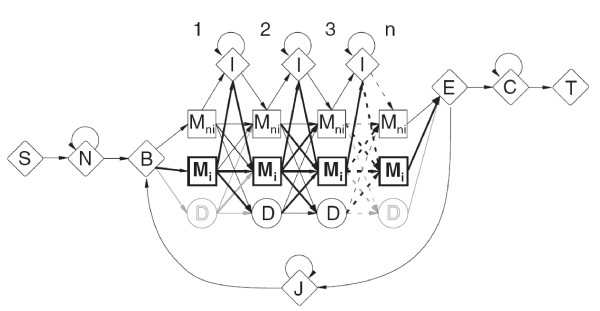
**Architecture of the interaction profile hidden Markov model**. The match states of the classical pHMM are split into non-interacting (*M_ni_*) and interacting (*M_i_*) match states.

Friedrich *et.al*. [25] proposed a model in which the interacting sites within protein domains are modeled by a modified pHMM, the ipHMM. The model architecture takes into account both structural information and sequence data. Every ipHMM is, like pHMMs, a probabilistic representation of a protein domain family. The architecture of the ipHMM follows the same restrictions and connectivity of the HMMER architecture [29], with one important exception: the match states of the classical pHMM are split into a non-interacting (*M_ni_*) and an interacting match state (*M_i_*), as shown in Figure 2. The new match state is provided with the same properties of a match state in the ordinary profile hidden Markov model architecture, *i.e.* these interacting match states are able to emit all amino acid symbols with probabilities, which are parameters to be fixed according to the training examples.

The parameters of an ipHMM are estimated using maximum likelihood based on a multiple sequence alignment of the member proteins in the domain family, incorporating the annotation on their binding sites - all residue positions are labeled with the corresponding interaction status (0 for not interacting and 1 for interacting). These trained ipHMMs thus encode relevant statistical information about the domain, especially pertaining to the interaction with other domains. Although the ipHMM was proposed by Friedrich *et.al*. for better identifying interacting domains from protein sequences, we show next that by extracting and selecting features from these models properly, we can build a powerful tool for PPI prediction.

### Negative examples

As explained in the Introduction, our method as a discriminative approach requires negative interaction examples, *i.e*. pairs of protein sequences whose interaction has been deemed very unlikely. But it is a well known problem that negative examples for protein-protein interaction prediction are hard to attain [30]. Given the current state of the art, when experimental methods like Y2H do not report an interaction between two proteins, it does not mean that the interaction is ruled out. In other words, the absence of a positive signal does not imply a negative signal. For this reason the confirmed negative examples in interacting domain families are very scarce. For our method, this situation is even worse, because we are looking for pairs of proteins that share an interacting interface, but even so, it is known that they do not interact - we do have one such case where a real negative example is known, and a detailed case study is given in subsection Case study of real negative examples.

To mitigate this problem, random sequences have been used as surrogate for negative examples, as reported in Aloy and Russell [31], which also uses protein sequences and 3D structure information for PPI prediction and will serve as a benchmark to compare with. In generating random sequences, we try to simulate the family membership of each sequence to its corresponding domain family. Specifically, our random generator (Figure 1, panel A) spawns sequences based on the consensus from the multiple sequence alignment of the training examples. Then, on top of the consensus sequence, we identify those positions where interacting residues are annotated in any sequence from the family, and overwrite that residue with a random amino acid (from a uniform distribution over the 20 amino acid alphabet). These sequences are aligned to their corresponding ipHMM, Fisher vectors are extracted from the alignment, and their concatenated vectors produce our negative examples (Figure 1, panel C).

### Fisher Scores

In this subsection we describe how to extract features from domain profiles, *i.e*. ipHMMs. Deriving and using Fisher scores from ipHMMs was first proposed in [32] and is reviewed here for the sake of being self-contained. Hidden Markov models, including ipHMMs, are probabilistic models that capture the collective features and characteristics of members in a family, in our case, proteins that contain the same domain. The typical tasks with a trained pHMM in general (with parameters *θ*) are to calculate a) the probability *P*(*x*|*θ*) of an unknown protein **x **to belong to the family, and b) how it aligns best with other members in the family [33]. While *P*(*x*|*θ*) is useful in telling if **x **is a member, the prediction based on this single value is susceptible to being false.

The false rate can be compounded (multiplied) if we use this method for predicting PPIs, as previously pointed out [5]. For example, domains A and B are known to be interacting with each other, and are profiled as ipHMMs respectively. If protein *x *is shown to possess domain A and protein *y *is shown to possess domain B, then we would predict that proteins *x *and *y *interact with each other. If the accuracy for predicting the membership of *x *and *y *is 60%, then the accuracy for predicting the *x *− *y *interaction will fall to 60% × 60% = 36%.

To prevent such a compounding effect of misclassification, we propose to use another classifier - a SVM - in tandem with the ipHMM. To do so, instead of using the probability *P *(*x*|*θ*) directly, we extract from this probability features that characterize both the domain profiles and the protein *x*. Specifically, we calculate the so called Fisher scores, defined as the derivatives of the probability for the query sequence *x *given the model *θ *with respect to particular parameters of the model. The use of Fisher scores in SVMs was first proposed by Jaakkola [26,27] in the context of detection of remote protein homologs, and was later adopted for other applications in bioinformatics, including discriminating signal peptides from proteins with a single transmembrane domain [34].

The benefits of using Fisher scores are many. Not only can they extract subtle and sensitive features as derivatives with respect to the model parameters, but also they allow for proteins with various lengths to be represented as vectors of the same length, where the vector components are derivatives and the vector dimension is determined by the number of model parameters that are deemed relevant and informative for the classification tasks. Having proteins represented as vectors of the same length is necessary for the SVM. Feature selection is natively supported because one can choose which parameters in the model to use for calculating the Fisher score. In this work we will focus on the emission probabilities of the match states.

If the probability of emitting amino acid $\tilde{x}$ from state $\tilde{s}$ is named $\theta_{\bar{x},\bar{s}}$, the Fisher score of the model with respect to $\theta_{\bar{x},\bar{s}}$ is therefore defined as

(1) $$\frac{\partial }{\partial \theta_{\tilde{x},\tilde{s}}}\log P(x|\theta )=\frac{\in (\tilde{x},\tilde{s})}{\theta_{\tilde{x},\tilde{s}}}-\in (\tilde{s})$$

where $\in (\tilde{s})=\sum_{x^{'}}\in (x^{'},\tilde{s})$ and the summation runs over the 20 different amino acids. The derivation of (1) is detailed in [27]. In this formula, $\in (\tilde{x},\tilde{s})$ can be seen as the expected posterior probability of visiting state $\tilde{s}$ and generating residue $\tilde{x}$ from that state. This expected value can be calculated, for any state $\tilde{s}$ and for any emitted amino acid $\tilde{x}$, from the posterior decoding matrix, which can be found efficiently using the forward and backward algorithms [33,29]. The literature denotes $\in (\tilde{x},\tilde{s})$ and $\in (\tilde{s})$ as the *sufficient statistics *for the parameter $\theta_{\bar{x},\bar{s}}$ in the model. For this reason we say that the *sufficient statistics *of the entire model are embedded in the Fisher scores.

Another type of Fisher score, the so called *constrained *Fisher score, was shown in [32] to behave very well in PPI prediction. In (1), *P *(*x*|*θ*) is the sum of contributions from all paths aligning *x *to the model. One could rather focus on the probability of the best path *s *that aligns *x *to the model, *P *(*s*|*x*, *θ*), and calculate the derivative of such *P *with respect to *θ*. This would give the constrained Fisher score, formally defined as:

(2) $$\frac{\partial }{\partial \theta_{\tilde{x},\tilde{s}}}\log P(s|x,\;\theta ).$$

### Feature selection with SVD

As discussed above, the Fisher score representation allows for choosing specific model parameters deemed relevant and informative in extracting features from the ipHMM. Intuitively, one feature selection scheme is that two sets of parameters are selected for comparison: emissions from the (non-interacting) match states (*M_ni_*) and emissions from the interacting match states (*M_i_*). While this feature selection is simple and useful, such a variety of attributes may not be feasible. Note that the average domain length in the datasets used in this study is 135 residues, leading to the same number of non-interacting match states (*M_ni_*) and interacting match states (*M_i_*). Multiplying this by 20, the number of amino acids emitted from each state, the Fisher score vector for a protein aligned against an ipHMM has a dimension of 5, 400 on average. For DDI prediction, the Fisher score vectors for a protein pair are concatenated, leading to a vector of dimension 10, 800. Feature selection using just the interacting match states will reduce the dimensions by half to 5, 400. This number still presents a challenge even for classifiers like SVMs which are known to be superior than others in handling a large number of attributes [35].

To further reduce the dimensions and select the most relevant and informative features, we use the singular value decomposition (SVD). SVD, and PCA (Principal Component Analysis), have been widely used in feature selection and dimensionality reduction. The underlying assumption is that the most relevant features may be composed as a linear combination of the given attributes, and the relevance of a feature is indicated by the corresponding eigenvalue for PCA or singular value for SVD: the higher the value the more relevant. Therefore, it is convenient to rank the singular values and choose the features with top singular values.

Note that SVD is chosen over PCA as the method for feature selection in this work primarily due to its lower computational cost: given *N *vectors of length 2, 700, PCA requires solving an eigenvalue problem for a 5, 400 × 5, 400 matrix, but for SVD the eigenvalue problem is solved for a *N *× 5, 400 matrix, with *N *being the number of training examples, which is typically less than 20. Another difference between PCA and SVD is that in PCA the covariance matrix has both its columns and rows corresponding to the original attributes whereas in SVD the matrix has its columns corresponding to the original attributes and its rows corresponding to training examples (proteins). This leads to a slightly different interpretation for the combinational coefficients in the two methods: in SVD the rows (proteins) play somehow more explicit roles in determining the combinational coefficients and thus how the features are composed from the given attributes; in PCA the training proteins would be "summed up" in calculating the covariance between attributes. In our testing, however, this does not give rise to any noticeable difference in the performance of the overall approach.

Let *D *= {*PO*; *NE*} be the training dataset containing positive and negative examples, a matrix of size *d *× *l*, where *d *= *p *+ *n *is the number of train vectors, *p *is the number of positive examples, and *n *the number of negative examples; *l *is the length of each vector. For our dataset (see section 2), *p *is usually around 10, and we make *n *= *p*. Experiments showed that making *n ***>***p *does not change the prediction results.

We could decide to extract the singular value decomposition from the entire dataset *D*. But interestingly enough, it was noticed that applying SVD only on the positive dataset is sufficient. And this is reasonable, because the informative signals in our data come from the sequence pairs whose 3D structure (and the interactions encoded in it) is known. The negative examples contain some of this information, plus random noise. Including the negative examples in the SVD would be wasteful, because the spectrum of the noise is flat, hence uninformative.

Let us formally define the singular value decomposition. The SVD of *PO *attempts to find two sets of orthonormal vectors, $\left\{{\hat{v}}_{1},{\hat{v}}_{2},\;\ldots ,\;{\hat{v}}_{r}\right\}$ and $\left\{{\hat{u}}_{1},{\hat{u}}_{2},\;\ldots ,\;{\hat{u}}_{r}\right\}$, where the former is an orthonormal basis for the row space of *PO*, and the latter is an orthonormal basis for the column space of *PO*. Also, *r *is the rank of *PO*, ${\hat{v}}_{i}$ is of length *l *and ${\hat{u}}_{i}$ is of length *p*. The SVD defines a very special relation between the ${\hat{v}}_{i}$ and the ${\hat{u}}_{i}$ vectors. Namely, for 1 ≤ *i *≤ *r*, *PO*·${\hat{v}}_{i}$ = *s_i_*·${\hat{u}}_{i}$, where *s_i _*is defined as the "*i *− *th *singular value". Therefore, if *V_PO _*= $\left[{\hat{v}}_{1}{\hat{v}}_{2}\ldots {\hat{v}}_{r}\right]$ and *U_PO _*= $\left[{\hat{u}}_{1}{\hat{u}}_{2}\ldots {\hat{u}}_{r}\right]$, and *S *is a diagonal matrix that contains the singular values in descending order, then *PO*·*V_PO _*= *U_PO_*·*S*. Since *V_PO _*is orthonormal, the positive dataset can be factorized as *PO *= *U_PO_*·*S*·$V_{PO}^{T}$, where *U_PO _*is of size *p *× *r *and *V_PO _*is of size *l *× *r*. The ${\hat{v}}_{i}$ vectors, which constitute a basis for the row space of *PO*, provide a means to reduce the dimensionality of each example vector in the following way: If we create a new matrix $V_{PO}^{RED}$ (*RED *stands for "reduced") of size *l *× *k*, where *k ***<***r *(only the strongest *k *base vectors have been maintained), we could project any example vector $\vec{e}$ of length *l *onto $V_{PO}^{RED}$ by simply doing ${\vec{e}}^{RED}={\left(V_{PO}^{RED}\right)}^{T}\cdot \vec{e}$. Clearly, ${\vec{e}}^{RED}$ is a column vector of length *k*. In other words, ${\vec{e}}^{RED}$ is the dimensionality reduced version of $\vec{e}$. These *k *new dimensions can be thought of as the features of each protein-protein interaction vector (be it positive, negative, a train or a test vector).

## 2 Data

In this section, we describe the data that are used in our study and how the training and testing sets are prepared.

### The Database of 3D Interacting Domains (3DID)

The training and testing examples in this study are obtained from a relational database of 3D Interacting Domains (3DID) [28] that contains a collection of (as of November 2009) 5, 175 domain-domain interactions (DDIs) in protein complexes with available information of three-dimensional structures via the Protein Data Bank. The 3DID criterion for physical interactions requires at least five contacts (hydrogen bonds, electrostatic or van der Waals interactions) between the two domains that have been detected, with a resolution of 10.0Å RMSD. Based on the structural information for the protein complexes at the atomic level provided by 3DID, we are able to determine which amino acids actually take part on the interaction and to construct an ipHMM. Each domain in the 3DID is characterized by training the model on all sequences that contain the domain using the procedure described by [24]. Consequently, each DDI is now modeled by the two corresponding ipHMMs for the two domains.

### Preparation of training and testing sets

For training and testing our method, we used a subset of DDIs extracted from 3DID. Each DDI is a family with interacting domains, *Dom. A *and *Dom. B*, whose members are *I *pairs of interacting proteins that have been found to physically interact, or in other words, there are *I *protein complexes with interacting proteins through the domain-domain interface and with distinct pdbid in the Protein Data Bank. We used DDIs with *I *between 10 and 11 and where the domain length (number of match states) is smaller than 300. These criteria look for families rich enough in information content for ensuring statistical robustness, but are not too big to avoid prohibitive processing times. With these filtering parameters, 121 DDIs were selected. For every protein sequence that is part of a single DDI, the 3DID database provides information to build binary vectors with the same length of the proteins and where the 1's indicate interacting amino acids. These vectors and the profile hidden Markov models of each domain, extracted from Pfam [24], are used to create ipHMMs for both domains.

To illustrate how we prepare the training and testing datasets, let us take a single DDI as an example. First, all the protein sequences in the DDI are aligned to their corresponding ipHMM (Figure 1, panels A and B). Alignments are obtained by posterior decoding (forward and backward algorithm). As explained in subsection Fisher Scores, from such alignments we can calculate the Fisher vectors. This algorithm can be efficiently executed through dynamic programming. As a result, each protein sequence can be numerically represented by the Fisher score vector. Positive examples are constructed by concatenating the Fisher vectors of interacting protein pairs (Figure 1, panel C). Negative examples are constructed as explained in subsection Negative examples. The SVD of the positive dataset is used to do feature selection on both, the positive and the negative train vectors (Figure 1, panel D). Now, with the complete training dataset reduced to *R^k^*, we can train a support vector machine (Figure 1, panel E). That concludes training. The last three stages of this pipeline give the name to our model: Fisher+SVD+SVM. We will use this name in the remainder of the paper.

For testing, a leave-one-out (LOO) strategy is followed. This guarantees that each positive example gets to be tested (predicted) once. In Figure 1, one interacting pair, reserved for testing (therefore not used in training), is used as a hypothetical query protein pair. The two sequences are aligned to their corresponding ipHMMs. For each DDI, 100 negative (random) examples are generated for testing. These sequences are also aligned to the ipHMMs. Fisher vectors are calculated, the entire test dataset is projected on *SVD^PO ^*(panel D), and finally each length *k *test example is classified using the SVM that was previously trained (panel E). The SVM provides the distance to the hyperplane for each test vector. These distances are used to calculate a histogram including the random sequences and the positive test example (panel F). An accurate classification would place this example far away from the negatives, with a high Z-score. Therefore, Z-scores can be used as a means of performance evaluation. We also report Area Under the ROC (AUC) curve results, calculated from the sorted list of distances to the hyperplane.

## 3 Results and Discussion

In this study, training and testing is implemented based on Matlab built-in functions for SVMs, namely *svmtrain *and *svmclassify*. Polynomial kernel with default parameters (order 3) was used. The number of iterative training and testing stages that are run per DDI is equal to the number of positive examples in the DDI. Note that each DDI is independently trained and tested. For feature selection, we used as many components of the singular value decomposition as needed to account for 80% of the variance in the positive dataset. This strategy showed outstanding experimental results, not only in speeding up the learning, but also in de-noising the information content of the Fisher vectors to improve prediction results.

### Performance evaluated by ROC score and Z-score

We tested our method on a dataset of 121 domain-domain interactions extracted from 3DID, prepared for training and testing as described in subsection Preparation of training and testing sets. Four different types of Fisher vectors can be created, as explained in subsection Fisher Scores: using the formula for the unconstrained Fisher score (1) or the formula for the constrained score (2), and in each of those cases, taking the derivative with respect to non-interacting match states or taking it with respect to interacting match states. Note that in each iteration our test set consists of one positive example and 100 negative examples. A perfect prediction from the SVM would assign to the positive example a distance to the hyperplane higher than the distances for all the negatives. To measure the actual performance, the receiver operating characteristic (ROC) sore is used. All testing examples are sorted in a descending order by their predicted distance to the hyperplane, and a moving threshold is used to scan through the sorted examples to make prediction: higher than the threshold is predicted as positive and others as negative. The ROC score is the normalized area under the curve (AUC) where the number of true positives is plotted as a function of the number of false positives. ROC score thus defined has a value ranged from 0 to 1, with the value 1 corresponding to the perfect prediction mentioned above, the value 0 to the worst performance, and a value 0.5 to a random classifier. In this study, we further evaluate the performance by Z-score. The predicted distances are used to produce a histogram, as schematically shown in Figure 1, panel F, where the horizontal axis is the predicted distance and the vertical axis is the number of examples receiving a specific predicted distance. The Z-score of the positive test example is calculated as

$$Z=\frac{D_{x}-\bar{D}}{\sigma }$$

Where *D_x _*is the predicted distance for example *x*, $\bar{D}$ is the mean for all predicted distances and *σ *is the standard deviation. Therefore, a higher Z-score indicates greater separation from the average, and hence higher confidence about the prediction.

Table 1 shows the results averaged over the 121 families. A total of 1, 255 distinct structures were predicted in this dataset. Of the four types of Fisher vectors used in testing, the *unconstrained *Fisher vectors calculated from non-interacting match states present the best performance, showing an outstanding average Z-score of 8.17 and an AUC of 93.48%. Specifically, under this scheme, 90 families show an average AUC greater than or equal to 90%, and 55 families have 100% accurate predictions: not a single structure in these families was incorrectly classified.

**Table 1:** Comparison of Z-scores and AUC scores using unconstrained and constrained Fisher vectors, averaged over **121 **DDIs

	Non-interacting	Interacting
**unconstrained**	Z-score = 8.17, AUC = 93.48%	Z-score = 7.09, AUC = 91.06%
**constrained**	Z-score = 4.32, AUC = 87.01%	Z-score = 6.94, AUC = 90.07%

**Figure 3:**
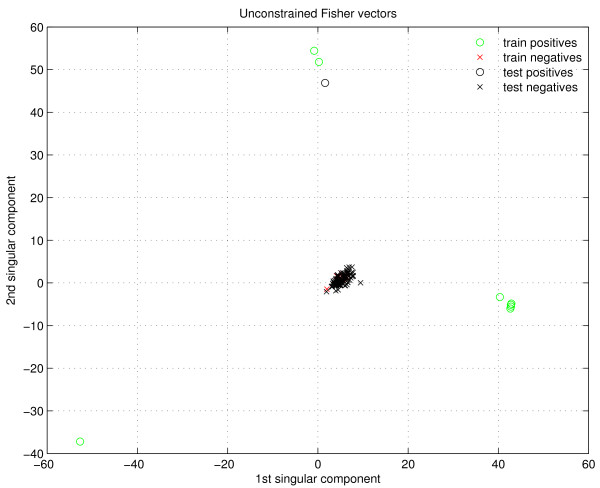
**First two SVD components of unconstrained Fisher vectors**. The domain-domain interaction family is AAA-Vps4_C, and the positive example being tested has pdbid 1xwi.

**Figure 4:**
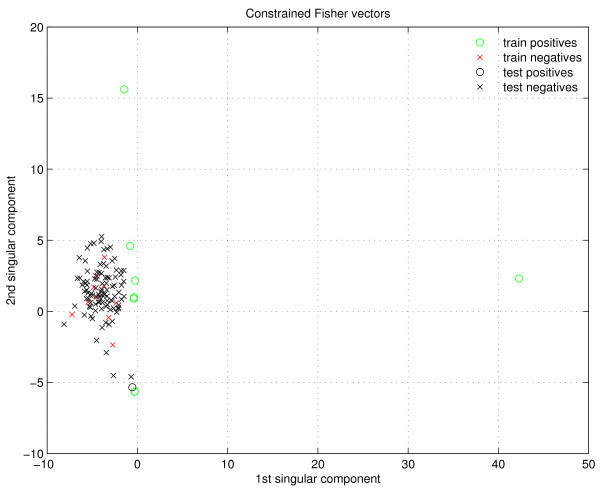
**First two SVD components of constrained Fisher vectors**. The domain-domain interaction family is AAA-Vps4_C, and the positive example being tested has pdbid 1xwi.

Unlike what is reported in [32], here we notice that the *unconstrained *Fisher score outperforms its *constrained *counterpart. We reason that this is caused by the way how the negative examples are generated (see subsection Negative examples). These negative examples are highly similar to the positive examples in positions where none of the three dimensional structures show residue-residue contacts. These positions are more numerous than interacting positions, therefore the similarity between positive and negative pairs can be high. Specifically, if the Fisher scores are calculated only along the most probable paths (*i.e*., *constrained*) for positives and negatives, the family membership could push both types towards a similar $\frac{\partial }{\partial \theta_{\bar{x},\bar{s}}}\log P(s|x,\;\theta )$, making their differentiation difficult. On the other hand, when using the *unconstrained *Fisher scores, the formula $\frac{\partial }{\partial \theta_{\bar{x},\bar{s}}}\log P(x|\theta )$ takes into account all the possible paths that align the sequences to their model, therefore the regions where the negative examples take on random residues can make a difference. This phenomenon is illustrated with a real example in the study of feature selection, presented in Figure 3 and Figure 4.

Figure 3 shows the power of SVD on the positive dataset for feature selection. The *unconstrained *Fisher vector is being used. In this example, taken from the domain-domain interaction AAA-Vps4_C, and where the positive protein pair being tested has pdbid 1xwi, the first two components of the SVD comprise more than 80% of the variance in the data. The plot shows these two components. Notice how the positive train examples (green circles) are spread in three well defined clusters, and all the negatives (red and black crosses) get mapped around (0, 0). The test positive example (black circle) lies near one of the positive clusters, and from the plot it is foreseeable that a SVM using polynomial kernel should be able to perfectly separate positives from negatives.

Figure 4 shows the same scenario, but when *constrained *Fisher vectors are used. In this case, the separation between positives and negatives is not very clear; plus, the test positive example lies very near the negatives. It would not be expected that a SVM were able to classify correctly this instance.

### Comparison with InterPreTS on Signaling, Cytokines-Receptors and Peptidases-Inhibitors families

We compared our method to an existing method using similar information for PPI prediction. In [31], a method called *InterPreTS *(Interaction Prediction through Tertiary Structure) [36] is developed. It takes a pair of query sequences and searches for homologues in a database of interacting domains of known three-dimensional complex structures. If one or more complexes are found, the query sequences are aligned to the closest homologues of known structure. Given the residue to residue matching produced by the alignment, and after identifying the residues that make atomic contacts in the complex of known structure, *InterPreTS *checks whether the query protein sequences preserve these interactions by means of empirical potentials. In other words, the pair of query sequences is scored for how well they preserve the atomic contacts at the interaction interface. Statistical significance of the potential interaction is estimated based on a background of random sequences. Another more recent technique in the same spirit of *InterPreTS *is *3D-partner*, although it also uses as input some other information such as contact residue interaction scores based on steric hydrogen bonds and electrostatic interactions [37].

Aloy and Russell [31] present prediction results running *InterPreTS *on domain-domain interaction families that can be grouped into three broad categories: Signaling, Cytokines-Receptors and Peptidases-Inhibitors families. We compared side by side the results of *InterPreTS *with the predictions that our method makes on the same dataset. The comparisons are summarized in Table 2. *InterPreTS *aligns the two query sequences to the closest homologues of known structure, and for each alignment it produces a Z-score based on how the empirical potentials of the alignment compare with the potentials from a background of 1, 000 random sequences. Since the homologues of known structure can be many, *InterPreTS *calculates Z-scores for each one of them and shows them in descending order (highest Z-score at the top). The *InterPreTS *column in Table 2 shows the averaged best Z-score over a family, that is, for each predicted complex structure, we only use the best score reported by *InterPreTS*. The table also shows the number of complexes found in each domain-domain family. According to [31], a Z-score ≥ 2.30 indicates a significance of the prediction of 99%, Z-score ≥ 1.30 indicates a significance of 90% and when Z-score **<**1.30 the two proteins are predicted not to interact in the same way as the known complex structure.

**Table 2:** Comparison between predictions made by InterPreTS and Fisher+SVD+SVM

category	Domain A	Domain B	# of distinct complexe	*InterPreTS *(avg. Z-score)	Fisher+SVD+SVM (avg. Z-score)
Signaling	RAS	Rho GAP	5	1.87	30.95
	RAS	Rho GDI	4	2.36	14.64
	G-alpha	Guanylate-cyc	15	3.70	22.95

Cytokines-Receptors	FGF	ig	6	1.01	24.55
	FGF	I-set	10	1.51	21.22

Peptidases-Inhibitors	Kringle	Trypsin	4	1.72	31.53
	Squash	Trypsin	9	1.28	10.23
	Kazal 2	Trypsin	4	0.73	30.64
	Peptidase M10	TIMP	6	0.61	31.35

On the other hand, when the Fisher+SVD+SVM approach is used to predict the interaction of a pair of query sequences, all the complex structures homologous to the sequences are used to build the model, and one single prediction is produced. The results shown in Table 2 for Fisher+SVD+SVM correspond to the *unconstrained *formula derived with respect to non-interacting match states, as this is the type of vector that consistently performs best. As before, a LOO strategy is followed for training and testing each family. For fairness, in these families we are also using, as *InterPreTS*, 1, 000 random sequences in testing.

Table 2 shows the outstanding performance of the Fisher+SVD+SVM method over *InterPreTS*, even for the Peptidases-Inhibitors families, in which *InterPreTS *performs poorly. Aloy and Russell [31] argue that this is because in these families the interactions occur via many main-chain to main-chain contacts (as opposed to main-chain to side-chain), and this negatively affects the usefulness of the empirical potentials. It seems that Fisher+SVD+SVM is able to circumvent this phenomenon and ultimately produce better predictions. One reason for this better performance of our method is, Fisher+SVD+SVM is able to produce one single, robust statistical model for the entire family of known 3D structures, whereas *InterPreTS *creates a different model for each complex. Another reason is, the SVM, being a binary classifier, reduces the variance of the test set by clustering together all the negative (random) examples on one side of the hyperplane, and pulling apart the positive example to the other side. A reduced variance would produce an increased Z-score, and that is exactly what we are observing.

### Case study of real negative examples

We also tested our method, along with *InterPreTS*, on protein pairs that contain the interacting domains but without actual interaction, thus called *real *negative examples. We mentioned before that this type of examples are difficult to find. In Aloy and Russell [31], however, one instance of such pairs is reported and studied: the Fibroblast growth factors (FGFs) and their homologues, the FHFs. FGFs mediate cell growth, differentiation, migration, and morphogenesis by binding to the extracellular domain of cell surface receptors, triggering receptor tyrosine phosphorylation and signal transduction [38]. FGF homologous factors (FHFs), however, do not bind to FGF receptors, instead they are associated with intracellular mitogen-activated protein (MAP) kinases.

**Figure 5:**
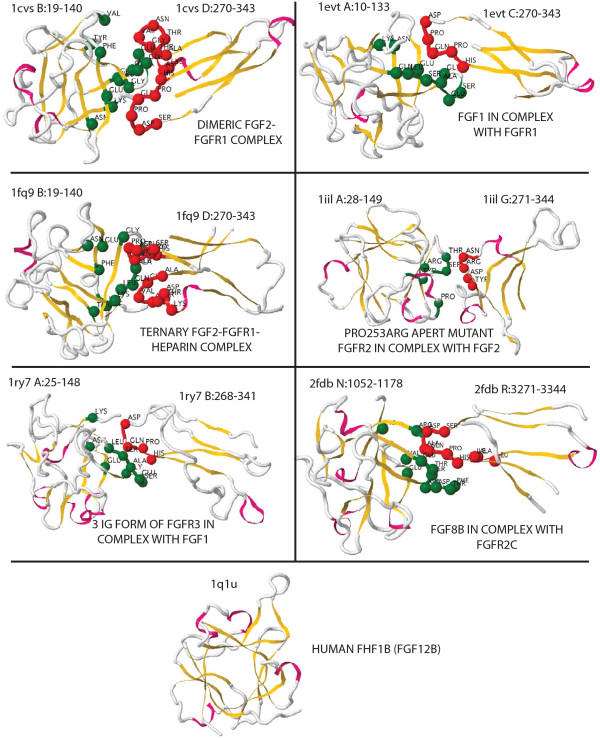
**Complexes that form the FGF-ig family**. Only the molecular structure of the domain interfaces is shown. The first three rows are the training complexes used to learn a model for the entire family. Green balls correspond to the **α**-carbons of the interacting amino acids in the FGF sequences. Red balls, likewise, correspond to **α**-carbons of interacting amino acids in the receptors. Yellow and pink ribbons show the secondary structure elements, *β*-sheets and *α*-helices respectively. The fourth row shows the secondary structure of one FHF sequence.

**Figure 6:**
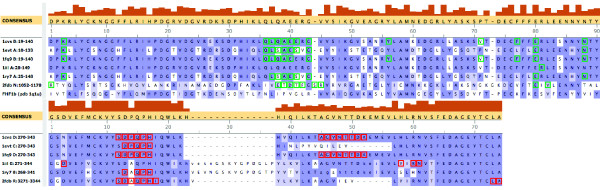
**Multiple sequence alignments of proteins in the FGF family and in the receptor (ig) family**. Part of the alignment for sequences in the FGF family is shown at the top (note that the whole alignment does not fit in the figure). Interacting positions are marked with green boxes. The homologous FHF1b sequence is also aligned. The bottom alignment shows the receptors, with red boxes marking the interacting residues.

We tested *InterPreTS *and our method with one FHF protein, the FHF1b (pdbid 1q1u), against all the FGF receptors found in the FGF-ig domain-domain interaction family. Figure 5 shows the molecular structure of the six complexes found in this family (first three rows), along with the FHF protein (fourth row). Red and green balls in the structures indicate the interacting positions in the FGF (green) and in its receptor (red). The MSAs in Figure 6 correspond to the sequences in the FGF family (top) and in the receptors family (bottom). The top MSA is shown up to position 90 for space constraints. Interacting positions have been marked with green and red boxes. The FHF1b sequence also appears as it is aligned to the FGF family.

For each structure in the FGF-ig family, we first run *InterPreTS *and Fisher+SVD+SVM on the positive example (the structure itself), and then we replace the FGF sequence with the FHF1b sequence, and leave the same receptor. This gives us six real negative pairs, on which we run both methods again. The results are listed in Table 3. It is expected for the Z-score of the positive example to be greater than the negative, and this is being the case in all structures for *InterPreTS*, and in all the structures but pdbid 2fdb for Fisher+SVD+SVM. However, the positive signal is somewhat weak and marginal in the *InterPreTS *results. On the other hand, for the Fisher+SVD+SVM scheme, not only the positive example receives very strong Z-score, but also the real negative is pushed to the very opposite side of the distribution, showing a strong ability for differentiation.

**Table 3:** Case study of the real negative examples.

	*InterPreTS*		Fisher+SVD+SVM	
pdbid	Positive Z-score	Negative Z-score	Positive Z-score	Negative Z-score
1cvs	1.02	0.47	31.21	−0.67
1evt	0.51	−0.91	27.76	−0.66
1fq9	1.03	0.51	31.21	−0.67
1iil	1.14	0.87	24.94	2.01
1ry7	1.08	0.66	28.79	−1.48
2fdb	1.28	0.10	3.38	4.30

Fibroblast Homolog Factor FHF1b was tested for interaction against all the receptors in domain-domain family FGF-ig

### Applicability of the Fisher+SVD+SVM method

We finish this section discussing the relation between the proposed computational method and the biological systems that concern the problem of DDI. The intuition driving this work is that structural information plays a key role in DDI prediction, and the success and limitations of any computational method is affected by how effectively we can extract, transfer and represent such information so that it can be better learned by a classifier to make more accurate predictions. So the success of our method comes from the following: a) leveraging the ipHMM to profile protein domains with structural input; b) using the Fisher scores to extract features that are most relevant to the profile; c) doing de-noising and feature selection through SVD. The limitations primarily come from two sources: 1) the reliability of the training data (for example, the interface residues can be determined on a higher resolution - the current resolution is 10.0Å RMSD); 2) assumption made by SVD that useful features can be obtained from linear combination of attributes, although in real world scenarios this can only be an approximation. Like any other computational method, this algorithm can complement the experimental approaches, which are typically more expensive and time-consuming. Particularly, due to its use of structural information regarding interface residues, the method can be used as a simulation tool in protein engineering, for example, to predict if and how the interaction of two proteins may change with mutations at certain residues and narrow the experiments to the most promising mutants.

## 4 Conclusions

In this work, we developed a computational method for predicting domain-domain interaction based on domain profiles. The method adopts a framework that is capable of combining in tandem the interaction profile hidden Markov models for domains and the support vector machines for domain-domain interaction prediction. The framework enables feature extraction and feature selection between the two tandem stages. By leveraging the interaction profile hidden Markov models trained on interacting protein domains whose structure is known, we are able to transfer the domain structural information to proteins that lack such information. We showed that the Fisher scores computed from alignments of protein sequences to the interaction profile hidden Markov models for domains offer more domain specific features characterizing protein sequences involved in interaction interfaces. The effect is more pronounced for non-interacting match states in ipHMMs, offering a powerful alternative when interacting residue information is not readily available. We also demonstrated that feature selection can play a key role in enhancing the signal-noise ratio for the next stage learning by the support vector machine. As a result of applying these techniques, our predictor is able to outperform another well known method based on the same sources of information. It is believed that by integrating the feature selection mechanisms with the learning process within a semi-supervised learning framework the method has the potential to be efficiently applied to genome wide prediction even with limited training data.

## Authors' contributions

LL conceived the initial ideas and supervised all aspects of the work. AJG designed and implemented the algorithms and the experiments. Both authors wrote and approved the manuscript.
